# *PGAP1*‐Related Encephalopathy in an Infant With Neurodevelopmental Delay: Novel Variant and Review of Literature

**DOI:** 10.1002/jdn.70044

**Published:** 2025-08-10

**Authors:** Savas Baris, Cuneyd Yavas

**Affiliations:** ^1^ Aydin Hospital Aydin Maternity and Children's Hospital Aydin Turkiye; ^2^ Department of Molecular Biology and Genetics Biruni University Istanbul Turkiye; ^3^ Biruni University Research Center (B@MER), Biruni University Istanbul Turkiye

**Keywords:** novel variant, *PGAP1* variant, spastic paraplegia‐67, whole exome sequence

## Abstract

Spastic paraplegia‐67, caused by a defect in glycosylphosphatidylinositol (GPI) biosynthesis, is an autosomal recessive neurodevelopmental disorder. It is characterized by dysmorphic features, spasticity, brain abnormalities, hypotonia, impaired intellectual development and speech difficulties. A 9‐month‐old girl was admitted to our clinic with a neurodevelopmental disorder, hepatomegaly, occasional vomiting, spasticity and hypotonicity. Whole exome sequencing (WES) was planned in the patient who had microcephaly, dysmorphic facial appearance, short and blunt fingers, a wide mouth, lacking physical sensation, dyskinetic movements, agenesis of the corpus callosum and cerebellar hypoplasia on MRI. In our study, we used whole‐exome sequencing, family segregation and bioinformatics to identify a homozygous 3 bp duplication in the GPI remodelling gene *PGAP1* (c.1226_1229dup p.(Val411Argfs*3) [NM_024989.4]) in a female patient with a neurodevelopmental disorder, encephalopathy and nonspecific autosomal recessive forms of intellectual disability (ARID). At least 26 genes are involved in the biosynthesis and remodelling of GPI junctions. Hypomorphic coding variants in seven of these genes have been reported to cause reduced expression of GPI‐associated proteins (GPI‐APs) on the cell surface and ARID. *PGAP1* gene variants are important in understanding GPI biosynthesis defects, which can lead to severe neurodevelopmental disorders like spastic paraplegia‐67. The identified homozygous variant (c.1226_1229dup) further expands the genetic spectrum of GPI‐related disorders and underscores the role of WES in diagnosing rare encephalopathies with dysmorphic features.

## Graphical abstract

This graphical abstract illustrates the comprehensive diagnostic approach, from next‐generation sequencing and bioinformatics to variant analysis and protein modelling, that led to the identification of a novel *PGAP1* gene variant in an infant with neurodevelopmental delay and encephalopathy.

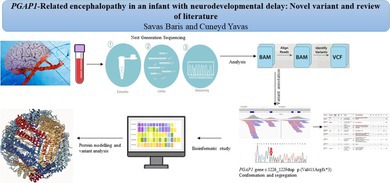

## 1 Introduction

Variants in proteins involved in glycosylphosphatidylinositol (GPI) anchor biosynthesis and remodelling pathway are associated with autosomal recessive forms of intellectual disability (ID) (Granzow et al. 2015; Murakami et al. 2014; Rachdi 1977). Individuals with homozygous or compound heterozygous pathogenic *PGAP1* variants exhibit a heterogeneous clinical presentation. Common findings reported in the literature include recessive inheritance patterns, along with spasticity, global developmental delay, microcephaly, eye/vision disorders, feeding difficulties, motor development delays, brain malformations, ID, delayed myelination and muscular hypotonia (Kettwig et al. 2016; Murakami et al. 2014; Rachdi 1977; Williams et al. 2015). Brain malformations are commonly associated with neuromotor developmental delays and a dysmorphic facial appearance (Balthazar 1977). Limb hypertonia and spasticity with hyperreflexia are common features seen in many patients (Fratto et al. 2024). The disease is characterized by general developmental delay beginning in infancy or childhood and progressive spasticity with tremor of the distal limbs and extensor plantar responses (Kamnasaran et al. 2010). The encoded protein is required for the production of GPI, which can bind to proteins, and this may be an important factor in the transport of GPI‐bound proteins from the endoplasmic reticulum to the Golgi (Bernat‐Silvestre et al. 2021; Hong et al. 2024; Lin et al. 2022).

GPI‐anchored proteins (GPI‐APs) play vital roles in neuronal development and cellular signalling. *PGAP1* encodes a key enzyme involved in the early remodelling of GPI anchors within the endoplasmic reticulum, and its dysfunction leads to defective maturation of GPI‐AP. Reported variants include missense, nonsense, splice‐site and frameshift variants, with varying clinical severity. However, the rarity of reported cases limits understanding of genotype–phenotype correlations. In this study, we describe a Turkish female infant with a novel homozygous *PGAP1* frameshift variant, c.1226_1229dup p.(Val411Argfs*3), identified through next‐generation sequencing. Clinical findings were supported by structural bioinformatic analyses predicting loss of functional protein domains. This report contributes new data to the expanding *PGAP1*‐related disorder spectrum and highlights the importance of functional assessment of novel variants.

## 2 Aim

The aim of this study was to investigate the genetic aetiology of a 9‐month‐old female patient presenting with neurodevelopmental delay, dysmorphic features, hypotonia and brain abnormalities, including agenesis of the corpus callosum and cerebellar hypoplasia. By employing whole‐exome sequencing, variant conformation and family segregation, we utilized to identify pathogenic variants in GPI biosynthesis‐related genes, particularly *PGAP1*, to confirm a diagnosis of spastic paraplegia‐67 and contribute to the understanding of genotype–phenotype correlations in this rare autosomal recessive disorder.

## 3 Materials and Methods

### 3.1 Genetic Studies

Genomic DNA was isolated from peripheral blood and subjected to next‐generation sequencing (NGS) using a commercial whole exome sequencing (WES) kit (Celemics, South Korea). Sequencing was performed on an Illumina MiSeq platform (San Diego, CA, USA) after library preparation and enrichment. Sequencing reads were aligned to the human genome reference (GRCh37/hg19) using BWA (Burrows‐Wheeler Alignment Tool), and BAM files were subsequently sorted, indexed and de‐duplicated using SAMtools and Picard. Although WES was performed, variant analysis was initially focused on a curated panel of 245 genes associated with spasticity. This panel included all exons, 5′ and 3′ untranslated regions and 25 base pairs of intronic flanking sequences for each gene. Raw sequencing data were processed using Trimmomatic, and postalignment processing included indel realignment and base quality score recalibration using GATK. Identified variants were annotated using databases and tools such as OMIM, PubMed, Franklin, InterVar, VarSome, ClinVar and Illumina BaseSpace Variant Interpreter. Variants with a population frequency > 0.5% were excluded, and pathogenicity was predicted using dbNSFP (SIFT, PolyPhen‐2, LRT, and MutationTaster). Rare variants were classified according to the ACMG/AMP guidelines.

All candidate variants were confirmed by Sanger sequencing, and segregation analysis was performed in the patient's family to determine inheritance status (Li et al. 2017; Richards et al. 2015). Genomic DNA from the patient and family members was amplified using primers flanking the *PGAP1* c.1226_1229dup region (forward: 5′‐TGCAAACATTGAAATTAGCTGGG‐3′ and reverse: 5′‐GCAGGCCATTCAAACATATCCT‐3′). PCR reactions were carried out under standard cycling conditions. The PCR products were first visualized by 2% agarose gel electrophoresis to confirm successful amplification and assess product size. Confirmed amplicons were purified and sequenced using an ABI 3500 Genetic Analyzer (Applied Biosystems, Foster City, CA, USA). Sequence analysis and variant confirmation were performed using SeqScape Software v4.0 (Applied Biosystems).

### 3.2 Bioinformatic Studies

The sequence data for *PGAP1* were obtained from NCBI and Uniprot databases. The sequences NM_024989.4 and Q75T13 were used as references. The sequence editing and comparisons were done with MegaXI and MutationTaster (Steinhaus et al. 2021; Tamura et al. 2021). The sequence alignment was made with MAFFT (v7.511) L‐INS‐i algorithm (Katoh et al. 2002). Protein homology models for the wt‐ and mt‐PGAP1 were created with I‐Tasser Software. The quality assessment of protein models was performed with Molprobity and QMEANDisco (Chen et al. 2010; Studer et al. 2020). Inositol compound 3D structure (CID_10228095) was retrieved from PubChem database and used. Wt and mt‐PGAP1 proteins were docked with inositol compound and functional bioinformatics study was performed with CB‐DCOK. PyMOL was used for processing and visualization of protein models (Liu et al. 2020; Yavas et al. 2025).

### 3.3 Patient and Study Permission

A 9‐month‐old girl with head retention, neurodevelopmental disorder, spasticity, indifference to the environment, somnolence, hepatomegaly and occasional vomiting was admitted to our clinic because of hypotonicity. Metabolic biotidinase enzyme was found to be low, but subsequent measurements were normal. The mother and father were first‐degree relatives. A 14‐year‐old sister was healthy. The patient presented with a range of clinical findings, including microcephaly, dysmorphic facial appearance, short, blunt fingers and a wide mouth. On further examination, lacking physical sensation and dyskinetic movements were noted. MRI scans revealed cerebellar hypoplasia and agenesis of the corpus callosum. On neurological follow‐up, tonic clonic contractions were detected several times. Permission for this study was obtained from the non‐interventional ethics committee of Aydın Obstetrics and Gynaecology and Paediatrics Hospital (Ethic Committee protocol no 2025/018).

## 4 Results

### 4.1 Genetic Findings and Genetic Analysis

**FIGURE 1:**
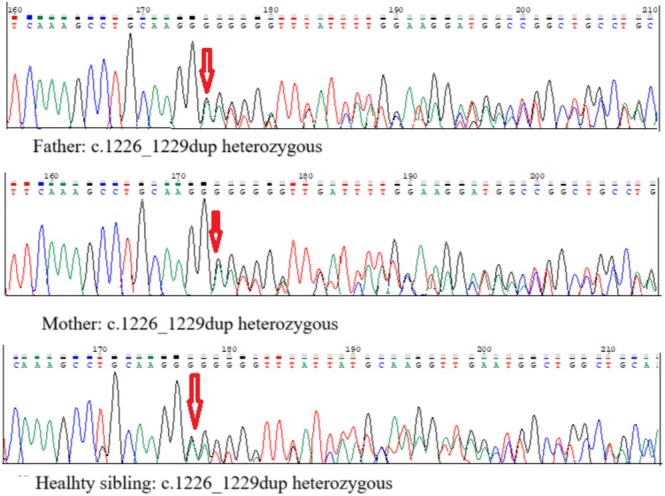
Segregation analysis of parents and healthy siblings.

As a result of the analysis, it was found that the patient was homozygous for the *PGAP1* gene c.1226_1229dup p.(Val411Argfs*3) variation in the 2q33.1 chromosome region. The variation is expected to cause a frameshift and early termination starting from position 411 in the 922 amino acid long Post‐GPI attachment to proteins 1 (PGAP1) protein encoded from the *PGAP1* gene. As a result of this variation, a region of approximately 511 amino acids including the GPI inositol‐deacylase beta‐sandwich domain functional/structural region between amino acids 462–595 is predicted to be lost in the truncated protein, thus affecting protein function (PGAP1_HUMAN, https://www.uniprot.org/uniprot/Q75T13). The variation has not been reported in any literature, but the fact that the variation causes early termination and was not detected in gnomAD exome scans suggests that this variation may have a clinical impact. Segregation analysis of the mother, father and sister revealed that c.1223_1229dup p.(Val411Alafs*3) variant in the *PGAP1* gene was detected as heterozygous in all three individuals shown in Figure 1. According to the ACMG variant interpretation guidelines, the variant classification c.1226_1229dup p.(Val411Argfs*3) in the *PGAP1* gene was based on the presence of a predicted loss‐of‐function (frameshift) effect in a gene known to cause disease through loss‐of‐function mechanisms, corresponding to criteria PVS1, and the variant was either absent or extremely rare in large population databases such as gnomAD, supporting criteria PM2. In the NGS data analysis performed considering the clinical information of the patient, it was concluded that this variant detected in the *PGAP1* gene is a likely pathogenic variant associated with the clinical findings of the patient. *PGAP1* gene c.1226_1229dup p.(Val411Argfs*3) variant results from in silico analysis are summarized in Table 1.

**TABLE 1:** Patogenicity of detected variant.

GENE	*PGAP1*
Transkript id	NM_024989.4
Dbsnp	Novel
Variant	c.1226_1229dup p.(Val411Argfs*3)
Variant locus	chr2:197750191 Exon 12
Variant type	Frameshit duplication‐nonsense
Seq.genomize	Not found
ClinVar	Not found
Domain	GPI inositol‐deacylase transmembrane GPI inositol‐deacylase beta‐sandwich
Conservation	Conserved
GnomAD exomes	Not found
LOVD	Not found
ACMG classification	Likely pathogenic
ACMG pathogenicity criteria	PVS1, PM2

Abbreviations: ACMG, The American College of Medical Genetics and Genomics; ClinVar, Clinically relevant variant; gnomAD, Genome aggregation database; seq.genomize, web based anutation programme‐turkish populastion variant database.

In the NGS analysis performed considering the clinical information and family history of the patient, genes associated with neuromotor developmental delay, dysmorphic face, hypotonia, vomiting, hepatomegaly and biotin deficiency in the Human Phenotype Ontology (HPO) database were primarily examined. As a result of the literature searches, it was concluded that the c.1226_1229dup p.(Val411Argfs*3) variant detected in the *PGAP1* gene among the genes associated with any disease in the OMIM database was a possible pathogenic variant associated with the clinical findings of the patient. Considering the age of the patient, re‐evaluation and follow‐up of the patient's clinical and other laboratory findings in terms of the clinical picture caused by pathogenic variants of the *PGAP1* gene are recommended. *PGAP1* gene pathogenic variants show recessive inheritance. Segregation analysis of the variant in all family members is recommended.

### 4.2 Bioinformatics and Genetic Analysis

**FIGURE 2:**
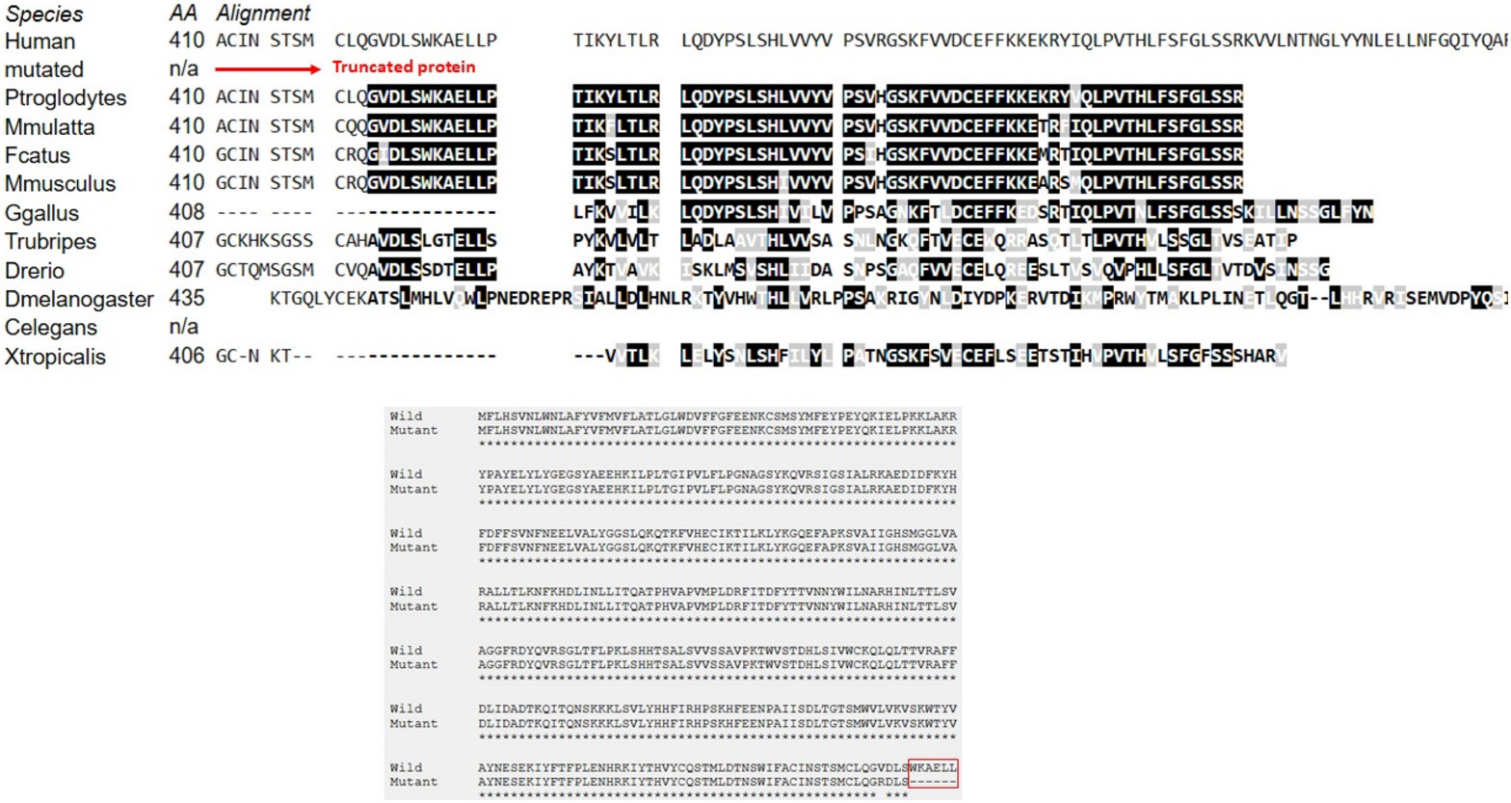
Evolutionary conservation of the affected protein region and alignment of wild‐type and mutant protein sequences. Multiple sequence alignment of the C‐terminal region of the protein across different species. The variant site in the human sequence is marked in red and results in a truncated protein product. Conserved residues are highlighted in black, indicating high sequence conservation among species. Alignment of wild‐type and mutant amino acid sequences showing the consequence of the variant on the protein structure. The mutant protein results in a premature stop codon leading to loss of the C‐terminal amino acids.

**FIGURE 3:**
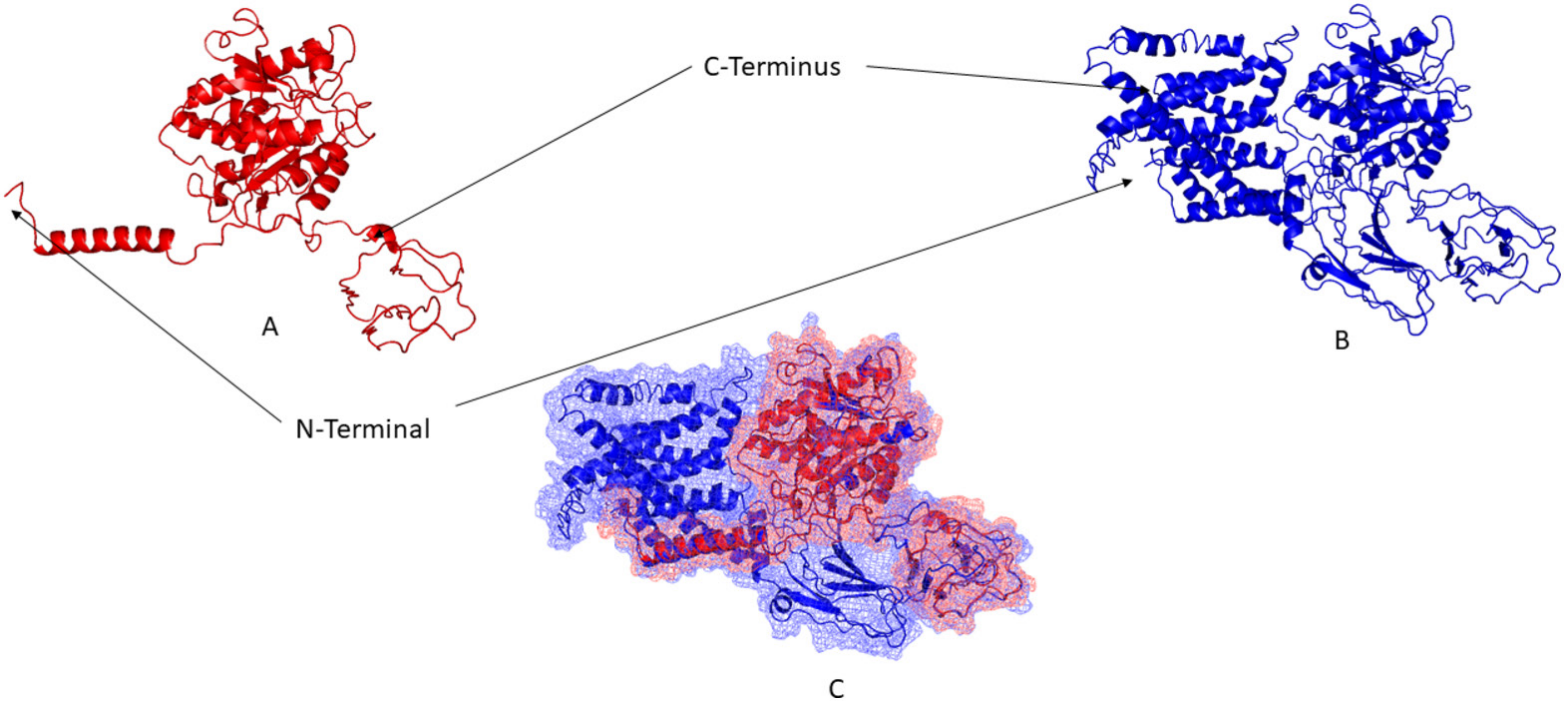
The image visualizes a prediction of protein folding by comparing (A) wild‐type (blue) and (B) mutant (red) conformations. The wild type (blue) shows the native, stable structure, while the mutant (red) highlights structural perturbations resulting from genetic variant. (C) Superimposed mesh representation aligns both structures, emphasizing their spatial differences. This overlay aids in visualizing deviations in backbone positioning, secondary structure elements or potential misfolding regions caused by the variant.

**FIGURE 4:**
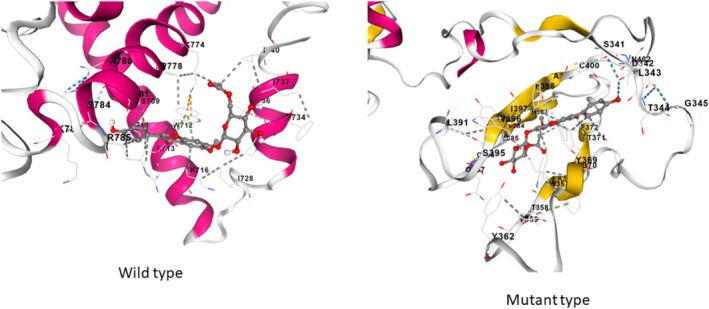
Comparative analysis of wild‐type and mutant‐type protein structures, focusing on amino acid changes induced by variant. The analysis identifies specific residue changes and predicts their structural or functional consequences.

**FIGURE 5:**
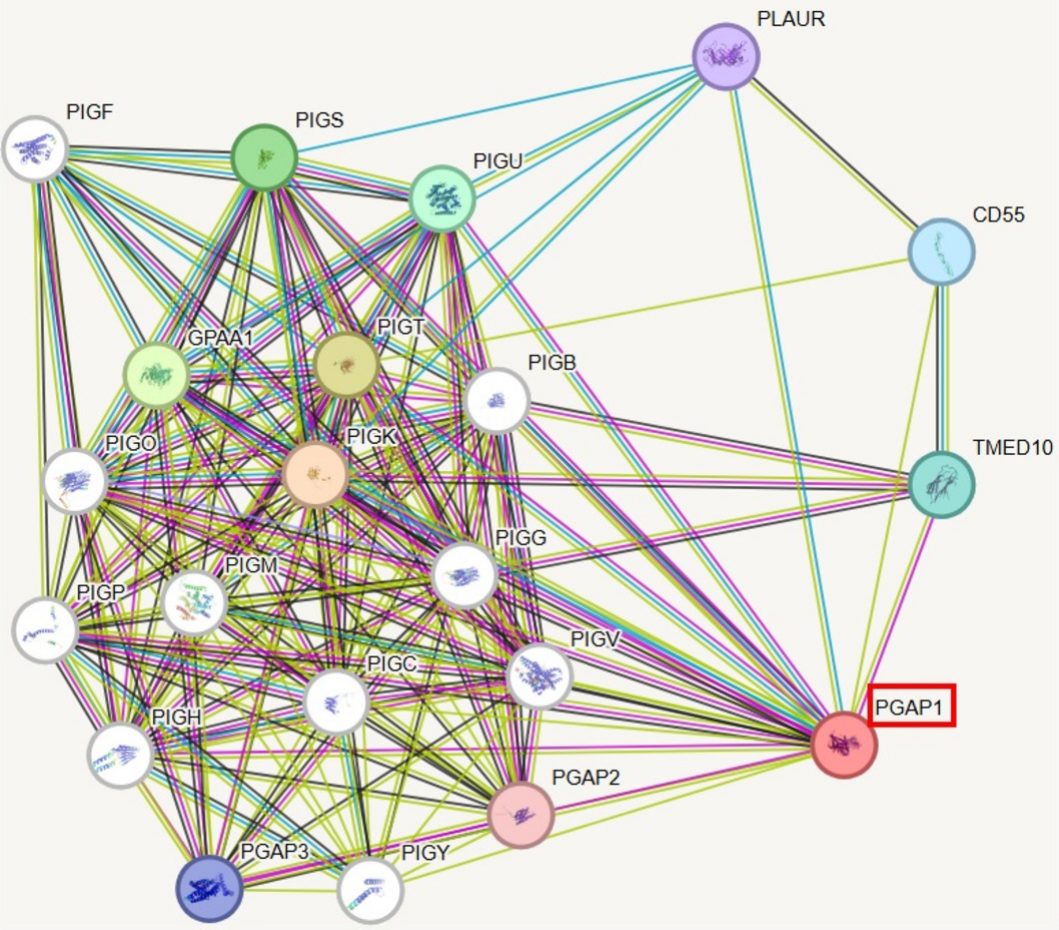
Interaction map of the PGAP1 protein. Edges represent protein–protein associations. Associations are meant to be specific and meaningful, that is, proteins jointly contribute to a shared function; this does not necessarily mean they are physically binding to each other. Each node represents all the proteins produced by a single, protein‐coding gene locus. Red node represent query protein. White node represent second shell of interactors. Represents the colours of the edges; black—coexpression, cyan—from curated databases, purple—experimentally determined, green—gene neighbourhood, red—gene fusions, navy blue—gene co‐occurrence, mustard green—textmining, blue—protein homology.

After the [NM_024989.4] c.1226_1229dup p.(Val411Argfs*3) variant, the length of *PGAP1* was shortened by 508 amino acids shown in Figure 2. The wt‐ and mt‐*PGAP1* proteins were modelled with I‐Tasser shown in Figure 3. The model quality scores were 0.51 ± 0.05 and 0.58 ± 0.05 for wt‐ and mt‐*PGAP1*, respectively. The model quality scores were within acceptable limits. After the variant of wt‐*PGAP1*, truncated protein formation was predicted as a result of premature termination by 4 amino acids shown in Figure 3. The interaction sites of the wild‐type and mutant proteins with inositol were identified, revealing distinct binding residues for each variant. The wild‐type protein interacts with the following amino acid residues: Ser709, Ser710, Trp712, Leu713, Lys716, Ile728, Pro730, Pro733, Phe734, Thr736, Ile737, Ile740, Ile741, Trp744, Thr745, Cys747, Gly748, Ala749, Thr771, Lys774, Asn775, Pro778, Val779, Asn780, Pro781, Lys782, Ser784, Arg785, Arg786, Ser787, Leu804, Ala809, Met863, Ala864, Ile865, Leu866, Gly867, Asn868 and Thr869. However, in the mutant protein, unlike the wild‐type protein, the residue interactions were significantly changed: Ser341, Asp342, Leu343, Thr344, Thr346, Ser347, Met348, Trp349, Val350, Leu351, Lys356, Trp357, Thr358, Tyr359, Tyr362, Glu366, Lys367, Tyr369, Phe370, Thr371, Phe372, Pro373, Leu374, His383, Val384, Tyr385, Cys386, Gln387, Trp396, Ile397, Phe398, Ala399, Cys400, Ile401, Asn402, Ser403, Cys407 and Leu413 shown in Figure 4. The protein functional relationship network of *PGAP1* was mapped using STRING shown in Figure 5. The pathogenic variant of the GPI inositol‐deacylase transmembrane region, which plays a role in the recognition of the inositol binding site, in the mutant PGAP1 protein may both cause it to fail to perform its function and affect the expression levels of many proteins in the protein interaction cascade that play an important role in neurological and global development.

## 5 Discussion

*PGAP1* encodes an enzyme responsible for the inositol deacylation of GPI‐AP, a crucial step in their biogenesis and quality control (Lin et al. 2022). This process takes place in the endoplasmic reticulum (ER) and is essential for the proper sorting, trafficking and function of GPI‐APs (Hong et al. 2024). *PGAP1* functions by removing the acyl chain from the inositol ring of the GPI anchor and converting triacylated GPI‐APs into diacylated forms, a remodelling step necessary for efficient ER export and the inclusion of GPI‐APs into Coat Protein II (COPII)‐coated vesicles for transport to the Golgi apparatus (Benabess and Myers 2025; Bernat‐Silvestre et al. 2021; Hong et al. 2024). Structurally, *PGAP1* is a multitransmembrane enzyme that employs a serine hydrolase‐like catalytic mechanism, with structural studies revealing a specialized substrate‐binding cavity that ensures specificity and prevents off‐target hydrolysis of membrane lipids (Bernat‐Silvestre et al. 2021; Hong et al. 2024). Additionally, PGAP1 plays a key role in quality control, as its slow enzymatic kinetics allow sufficient retention time in the ER for the proper folding and sorting of GPI‐APs, thereby serving as a checkpoint in their biogenesis (Hong et al. 2024).

Since *PGAP1* does not have an existing NMR or X‐ray structure, the wild type and mutant type *PGAP1* protein models were modelled using deep learning algorithms. The p.(Val411Argfs*3) variant caused shorter protein formation and loss of functional domain regions compared to wild‐type GPI inositol‐deacylase protein shown in Figures 2 and 3. The reduction of the regions required for the processing of the variant, loss of active sites, may trigger changes in protein stability and functional properties. p.(Val411Argfs*3) variant caused a frameshift after the conversion from valine to arginine at position 411 and caused premature termination after three amino acids shown in Figure 2. According to the results of the docking studies, the binding site of inositol changed after the variant shown in Figure 4. These findings suggest that the variant alters the interaction network, potentially affecting the protein's functional properties. It is thought that homozygous or combined heterozygous pathogenic variants in *PGAP1* may have an impact on the function of Post‐GPI Attachment to Proteins (Bernat‐Silvestre et al. 2021; Fujita et al. 2006). Since the variant enzyme activity detected in our study prevents the formation of active sites, both the results of our clinical study and our bioinformatic studies suggest that this variant is effective in GPI‐AP maturation.

Variants in the *PGAP1* gene have been identified as causative factors for a range of phenotypes, primarily associated with neurodevelopmental disorders. These variants include compound heterozygous variants, such as deletions (c.274_276del (p.(Pro92del)) and c.921_925del (p.(Lys308Asnfs*25))), which have been reported in patients with cerebral visual impairment (CVI) and ID. Additionally, the c.914C>T (p.(Ala305Val)) variant was identified but found to have no functional impact. Null variants, such as NM_024989.3:c.589_591delCTT (NP_079265.2:p.Leu197del), have been shown to impair GPI‐anchor maturation, leading to abnormal GPI‐AP structures (Bosch et al. 2015). Severe encephalopathy, characterized by epilepsy, spasticity and facial dysmorphism, has also been linked to *PGAP1* pathogenic variants (Benabess and Myers 2025). In this study, we demonstrated that the homozygous nonsense likely pathogenic *PGAP1* gene p.(Val411Argfs*3) variant in a patient was caused by both genetic diagnostic tools and bioinformatic tools.

Literature reviews related to the clinical correlation of the study are summarized (Benabess and Myers 2025; Fratto et al. 2024; Granzow et al. 2015; Kettwig et al. 2016; Murakami et al. 2014; Novarino et al. 2014; Williams et al. 2015). In the examination, as in other literature publications, the clinical findings arising from loss‐of‐function homozygous variants in the *PGAP1* gene are consistent with our case shown in Table 2. Our patient is a 9‐month‐old Turkish female with a homozygous c.1226_1229dup p.(Val411Argfs*3) variant in the *PGAP1* gene. Clinically, she presents with agenesis of the corpus callosum and cerebellar hypoplasia, accompanied by developmental delay, speech delay, growth retardation, microcephaly and seizures. This phenotype is comparable to previously reported cases, particularly those described by Novarino et al. and Williams et al. where patients also exhibited corpus callosum agenesis, cortical sulcal prominence and white matter reduction (Novarino et al. 2014; Williams et al. 2015). In contrast, one of the cases reported by Murakami et al. had normal brain imaging, while the other showed only mild atrophy, indicating variability in neurological involvement (Murakami et al. 2014). Similarly, patients described by Williams et al. and Granzow et al. displayed cerebral abnormalities and developmental delay, consistent with our patient's presentation (Granzow et al. 2015; Williams et al. 2015). Notably, the presence of microcephaly and prenatal anomalies in our proband distinguishes her from some of the reported cases, suggesting a broader clinical spectrum.

**TABLE 2:** Overview of *PGAP1*‐related phenotypes and variants reported in the literature.

Reference	Nationalty	Patient count	Gender	Age	Variant	Z	Brain abnormalities	DD	SPC	SD	GR	MC	PA	S	FA
Proband	Turkiye	1	F	9/12	c.1226_1229dup p.(Val411Argfs*3)	HM	Corpus callosum agnesis, cerebellar hipolplazi	+	+	+	+	+	−	+	Mild
Benabess and Myers	Pakistani	1	M	19	c.(1861+1_1862+2_1952+1_1953‐1)del	HM	Brain volume loss	+	+	+	+	+	+	+	Mild
Fratto et al.		1	F	0.23	c.832G>T p.(Gly278Trp) and c.1910A>G p.(Tyr637Cys)	Comp HT	Thinned spinal cord with borderline T2‐hyperintensities of the periventricular white matter	+	+	NA	+	NA	NA	NA	NA
Bosch et al		1	M	8	c.274_276del p.(Pro92del) and c.921_925del p.(Lys308Asnfs*25)	Comp HT	Normal	+	+	+	+	NA	NA	−	Mild
Murakami et al.	Syrian	2	1 M/1F	0.2/4	c.589_591del p. (Leu197del)	HM	Brain atrophy/Normal	+	−	+/−	+	NA	.+/−	Mild	NA
Novarino et al.		2	M	0.6/0	c.1952+1G>T	HM	Prominent cortical sulci, widened sylvian fissures, corpus callosum agnesis, vermis hypoplasia, defective myelination	+	+	+	+	NA	NA	−	NA
Williams et al.		1	M	3	c.1572 T>A (p.Tyr524*) and c.1396C>T p.(Gln466*)	Comp HT	Thinning of thecorpus collosum, diminished white matter, prominence of the right posterior Sylvian fissure, Prominent cortical sulci and widened sylvian fissures	+	+	+	+	+	NA	.−/+	Mild
Granzow et al.		2	M/F	0.8/4	c.1090‐2A>G	HM	Normal	+	−	−	+	+	NA	−	−
Kettwig et al.	Caucasian	2	M	0.2	c.334_335insA p.(Arg112fs*5) and c.1173G > C p.(Leu391Leu)	Comp HT	Frontal accentuated brain atrophy and significant delayed myelination	+	−	−	+	+	NA	.−/+	Mild

Abbreviations: Comp HT, compound heterozygous; DD, developmental delay; FA, facial anomaly; FD, feeding difficulities; GR, growth retardation; HM, homozygous; MC, microcephaly; PA, prenatal anomalies; SD, speech delay; S, seizure; SPC, spasticity; Z, zygosity.

Frameshift variants, such as the one observed in our proband (p.(Val411Argfs*3)), as well as splice‐site (c.1952+1G>T, c.1090‐2A>G) and nonsense variants (p.(Tyr524*), p.(Gln466*)), are generally associated with significant neurological impairments, including brain malformations, developmental delay and seizures. For instance, Novarino et al. (2014) reported splice‐site variants resulting in corpus callosum agenesis and cerebellar hypoplasia, mirroring our patient's neuroimaging findings. In contrast, Fratto et al. (2024) described compound heterozygous missense variants associated with milder findings such as borderline white matter changes and spinal cord thinning but without gross structural anomalies. This disparity suggests that frameshift, nonsense and splice‐site variants may have a more deleterious impact on *PGAP1* function, leading to more severe clinical outcomes.

The proband carries a homozygous frameshift variant predicted to result in a premature stop codon and consequent loss of protein function. This variant type is consistent with those reported in patients with severe phenotypes, including those with nonsense (Williams et al. 2015) and splice‐site (Novarino et al. 2014) variants, where overlapping features such as structural brain anomalies, developmental delay and seizures were frequently observed. These findings support the notion that loss‐of‐function variants in *PGAP1* are associated with a recognizable neurodevelopmental syndrome. However, in‐frame deletions (p.(Leu197del)) and missense variants have been associated with milder or more variable phenotypes, potentially reflecting less disruptive effects on protein function. Interestingly, some splice‐site variants, such as those described by Kettwig et al., were associated with normal brain imaging, emphasizing the importance of not only the variant type but also its location and impact on gene splicing or protein structure (Kettwig et al. 2016). The proband's phenotype aligns with the more severe end of the *PGAP1*‐related disorder spectrum.

In this study, we present a novel homozygous frameshift variant, p.(Val411Argfs*3), in the *PGAP1* gene, associated with a severe neurodevelopmental phenotype characterized by corpus callosum agenesis, cerebellar hypoplasia, developmental and speech delays, microcephaly and seizures. Structural modelling and docking analyses revealed significant truncation of the PGAP1 protein, with consequent loss of key functional domains and altered inositol‐binding capacity, supporting the pathogenic nature of the variant. Comparative analysis with the literature suggests that frameshift, nonsense and splice‐site variants are consistently associated with more severe clinical presentations, whereas missense and in‐frame deletions may result in milder or variable phenotypes. Our findings contribute to the expanding clinical and molecular landscape of *PGAP1*‐related disorders and emphasize the importance of integrating genomic and structural data to understand the consequences of novel genetic variants.

## Author Contributions

The study was planned by S.B. and C.Y. The clinical examinations of the participants were evaluated by S.B. All sequencing was performed by S.B. Analyses and bioinformatic studies were performed by C.Y. The primary manuscript was written by S.B. and C.Y. Figures and tables were prepared by C.Y. All authors have read and approved the final manuscript.

## Consent

Informed consent was obtained from all subjects involved in the study.

## Conflicts of Interest

The authors declare no conflicts of interest.

## Data Availability

All data supporting the findings of this study are available from the corresponding author upon reasonable request.
